# Prehospital resuscitative endovascular balloon occlusion of the aorta during long-distance ground transport for traumatic liver injury: A case report

**DOI:** 10.1016/j.tcr.2026.101407

**Published:** 2026-06-26

**Authors:** Hiroki Satou, Akihito Tampo, Motoi Okada

**Affiliations:** a Department of Emergency Medicine, Asahikawa Medical University, 2-1-1-1 Midorigaoka-Higashi, Asahikawa, Hokkaido, 078-8510, Japan

**Keywords:** Resuscitative endovascular balloon occlusion of the aorta, Prehospital care, Long-distance transportation, Rural trauma system, Decision-making

## Abstract

**Background:**

Resuscitative endovascular balloon occlusion of the aorta (REBOA) is an established adjunct treatment of hemorrhagic shock caused by subdiaphragmatic bleeding. However, evidence for its use in prehospital settings and long-distance transportation in remote locations remains limited. Herein, we report a case of hemorrhagic shock secondary to traumatic liver injury, for which prehospital REBOA was successfully employed.

**Case presentation:**

A 41-year-old man sustained multiple traumatic injuries after being run over by heavy machinery. The patient was diagnosed with hemorrhagic shock resulting from a grade IIIb liver injury. Owing to limited local resources and the anticipated clinical deterioration during long-distance transportation to an emergency medical center, a physician-staffed ground ambulance was dispatched. During which the hemodynamic status of the patient deteriorated, tracheal intubation and REBOA were performed in the ambulance. Partial aortic occlusion was initially applied, followed by complete occlusion when cardiac arrest was imminent. On arrival in the operating room, the patient underwent massive blood transfusion, damage-control laparotomy with perihepatic packing, and subsequent transcatheter arterial embolization. No REBOA-related complications occurred. The patient was discharged from the intensive care unit on hospital day seven.

**Conclusion:**

Prehospital REBOA may serve as an effective bridge for definitive hemorrhage control during long-distance transportation in resource-limited regions. Successful implementation requires experienced trauma teams and coordinated system-level management.

## Highlights

• Evidence for prehospital REBOA during long-distance transport is limited.
• Prehospital REBOA was performed during a 51-km ground transport.
• Stepwise aortic occlusion from partial to complete aided shock management.
• Treatment success required a coordinated trauma system and experienced team.
• Prehospital REBOA can serve as a bridge to definitive hemorrhage control.

## Introduction

Hemorrhage remains the leading cause of preventable trauma death, emphasizing the importance of rapid hemorrhage control from the prehospital phase until definitive source control [1]. Recent developments in trauma systems, including hybrid emergency room models, have improved outcomes in severely injured patients. However, these resources are not universally available, particularly in geographically vast or resource-limited regions [2], [3].

In northern Hokkaido, from where this case is reported, long transport distances and limited access to trauma-specialized facilities necessitate early physician-led interventions and strategic transport planning. Herein, we report a case of traumatic liver injury complicated by hemorrhagic shock in which prehospital resuscitative endovascular balloon occlusion of the aorta (REBOA) was successfully employed during long-distance ground transportation, enabling a safe transition to definitive hemorrhage control in a resource-limited trauma system.

## Case presentation

During winter, a 41-year-old man was struck and run over by heavy machinery and subsequently transported to a local hospital. On arrival, the patient presented a patent airway; respiratory rate, 30 breaths per minute; oxygen saturation, 97% on supplemental oxygen; blood pressure, 114/93 mmHg; heart rate, 64 beats per minute; peripheral coldness and pallor; and a Glasgow Coma Scale (GCS) of 15.

Contrast-enhanced computed tomography revealed a grade IIIb liver injury involving the anterior and posterior segments of the right hepatic lobe, along with hemoperitoneum (Fig. 1). Additional findings included a right pneumothorax, left pulmonary contusion, right scapular fracture, right humeral fracture, multiple right rib fractures, and bilateral pubic fractures. Laboratory findings demonstrated metabolic acidosis with a pH of 7.279 and a lactate level of 37 mg/dL, suggesting early hemorrhagic shock.

**Fig. 1:**
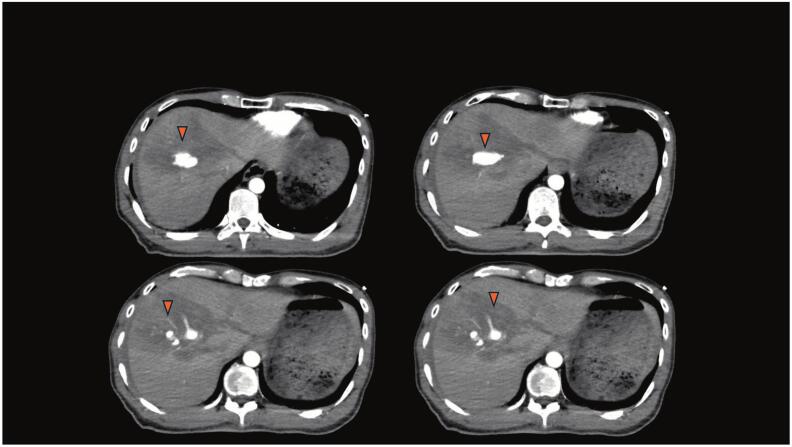
Contrast-enhanced computed tomography. This revealed a grade IIIb liver injury with hemoperitoneum (arrow).

As the referral hospital lacked blood components and a trauma surgical unit, an interfacility transfer to a secondary center, approximately 51 km away, was planned. Helicopter transport was unavailable due to the adverse weather and, thus, a physician-staffed ground ambulance was dispatched.

During transfer of the patient to the tertiary hospital, the patient's hemodynamic status deteriorated with diminished femoral arterial pulsation and decreased level of consciousness (GCS E1V2M1). Tracheal intubation was performed and a REBOA catheter (Rescue Balloon®-ER; Tokai Medical Products, Inc., Aichi, Japan) was inserted through the common femoral artery, and partial aortic occlusion was initiated. However, complete occlusion was performed later due to impending cardiac arrest prior arrival to the hospital.

On arrival, the patient was transported directly to the operating room (Fig. 2). Massive transfusion was initiated, followed by a laparotomy for damage-control, with perihepatic gauze packing. Subsequent angiography demonstrated active extravasation from branches of the superior mesenteric and gastroduodenal arteries, both of which were successfully embolized. The balloon of the REBOA catheter was deflated after hemodynamic stabilization was achieved and no REBOA-related ischemic or vascular complications were observed. The patient was discharged from the intensive care unit on postoperative day 7 and eventually transferred to the referral hospital following recovery.

**Fig. 2:**
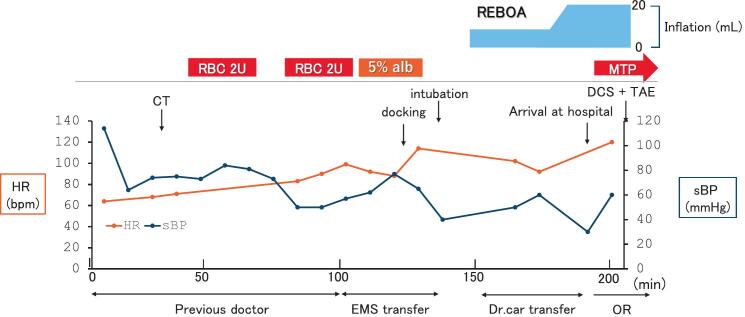
Clinical course. This timeline shows the progression from injury to damage control surgery, including prehospital REBOA placement, surgical intervention.

## Discussion

Hemorrhage remains the leading cause of preventable trauma deaths; thus, early hemorrhage control is critical for improving outcomes [1], [4], [5], [6], [7]. In situations where long-distance transport to definitive care is unavoidable, timely intervention during the prehospital phase may impact considerably on patient survival [8], [9].

This case demonstrates the feasibility and potential benefits of prehospital REBOA during long-distance ground transport for patients with traumatic hemorrhagic shock. Although the use of REBOA has expanded across hospitals, evidence for its use in prehospital settings-particularly during extended interfacility transport remains limited [10], [11], [12], [13], [14], [15]. In the present case, prehospital REBOA was considered essential for maintaining hemodynamic stability while the patient was transferred to definitive hemorrhage control.

A key aspect of this case was the stepwise REBOA strategy. Partial aortic occlusion was initially employed to balance hemorrhage control with the risk of distal ischemia, which then turned into complete occlusion only when impending cardiac arrest was suspected. This approach aligns with the emerging concepts in REBOA management; recent reports suggest that partial occlusion may reduce ischemic complications while preserving proximal perfusion [16]. Ongoing multicenter trials, including the PROMPT trial [17], are expected to further elucidate the optimal REBOA strategies and indications, including their roles in prehospital settings.

Beyond the technical feasibility of REBOA, this case highlights the importance of the trauma system organization and leadership. Effective leadership influences team performance and patient outcomes in high-acuity trauma settings [18]. In Japan, hybrid emergency room systems have been increasingly adopted and are associated with improved trauma outcomes; however, such infrastructure is not universally available. In geographically vast and resource-limited regions, such as northern Hokkaido, long-distance transport to emergency medical centers is often unavoidable, and initial care facilities may lack the resources required for definitive hemorrhage control. Under these circumstances, early physician-led interventions and optimal transportation planning are essential. In this case, decisive command during prehospital deterioration enabled timely escalation from partial to complete REBOA, which was critical during prolonged transport.

The successful outcome in this case was achieved not solely by implementing REBOA, but through coordinated trauma management, including rapid decision-making, appropriate escalation of resuscitative strategies, and a seamless transition to definitive surgical and endovascular hemostasis. The use of a physician-staffed ground ambulance enabled the rapid dispatch of experienced trauma specialists and essential equipment, thereby mitigating the limitations imposed by geographical and environmental conditions.

This case supports the notion that prehospital REBOA, when properly integrated into a coordinated trauma system, can serve as an effective bridge to definitive hemorrhage control during long-distance transport. To improve trauma outcomes in geographically constrained regions, establishing standardized protocols, maintaining continuous team training, and strengthening system-level coordination is crucial [19].

## Conclusion

Prehospital REBOA may be a valuable adjunct for managing hemorrhagic shock during long-distance transport in resource-limited trauma systems. However, the safe and effective use of REBOA requires experienced teams, clear indications, and a coordinated trauma management.

## CRediT authorship contribution statement

**Hiroki Satou:** Conceptualization, Writing – original draft, Writing – review & editing. **Akihito Tampo:** Conceptualization, Writing – original draft, Writing – review & editing. **Motoi Okada:** Writing – review & editing.

## Ethics approval and consent to participate

This report was conducted in compliance with the tenets of the Declaration of Helsinki. Written informed consent was obtained from the patient for the publication of this case report and the accompanying images.

## Funding

This research did not receive any specific grant from funding agencies in the public, commercial, or not-for-profit sectors.

## Declaration of competing interest

The authors declare that they have no competing interests.

## Data Availability

No datasets were generated or analyzed during the current study.

## References

[1] Kauvar D.S., Lefering R., Wade C.E. (2006). Impact of hemorrhage on trauma outcome: an overview of epidemiology, clinical presentations, and therapeutic considerations. J. Trauma.

[2] Kinoshita T., Yamakawa K., Matsuda H., Yoshikawa Y., Wada D., Hamasaki T. (2019). Survival benefit of a novel trauma workflow that includes immediate whole-body computed tomography, surgery, and interventional radiology, all in one trauma resuscitation room: a retrospective historical control study. Ann. Surg..

[3] MacKenzie E.J., Rivara F.P., Jurkovich G.J., Nathens A.B., Frey K.P., Egleston B.L. (2006). A national evaluation of the effect of trauma-center care on mortality. N. Engl. J. Med..

[4] Morrison J.J., Rasmussen T.E. (2012). Noncompressible torso hemorrhage: a review with contemporary definitions and management strategies. Surg. Clin. North Am..

[5] Kisat M., Morrison J.J., Hashmi Z.G., Efron D.T., Rasmussen T.E., Haider A.H. (2013). Epidemiology and outcomes of non-compressible torso hemorrhage. J. Surg. Res..

[6] Beck B., Bray J.E., Cameron P. (2017). Resuscitation attempts and duration in traumatic out-of-hospital cardiac arrest. Resuscitation.

[7] Morrison J.J., Yapp L.Z., Beattie A., Devlin E., Samarage M., McCarrer C. (2016). The epidemiology of Scottish trauma: a comparison of pre-hospital and in-hospital deaths, 2000 to 2011. Surgeon.

[8] Hsieh S.L., Hsiao C.H., Chiang W.C., Shin S.D., Jamaluddin S.F., Son D.N. (2022). Association between the time to definitive care and trauma patient outcomes: every minute in the golden hour matters. Eur. J. Trauma Emerg. Surg..

[9] Holcom J.B. (2018). Transport time and preoperating room hemostatic interventions are important: improving outcomes after severe truncal injury. Crit. Care Med..

[10] Hitoshi A., Richard H., Hideaki G. (2022). On-site placement of resuscitative endovascular balloon occlusion of the aorta in a hemorrhagic shock patient: a successful endeavor involving long-distance air transport. Am. J. Emerg. Med..

[11] Marsden M., Lendrum R., Perkins Z., Davenport R.A. (2025). REBOA for remote damage control resuscitation and the race against time. Curr. Opin. Anaesthesiol..

[12] Yamamoto R., Alarhayem A., Muir M.T., Jenkins D.H., Eastridge B.J., Shapro M.L. (2022). Gaining or wasting time? Influence of time to operating room on mortality after temporary hemostasis using REBOA. Am. J. Surg..

[13] Broome J.M., Ali A., Simpson J.T., Tran S., Tatum D., Taghavi S. (2022). Impact of time to emergency department resuscitative aortic occlusion after noncompressible torso hemorrhage. Shock.

[14] Brenner M., Bulger E.M., Perina D.G. (2018). Joint statement from the American College of Surgeons Committee on Trauma (ACS COT) and the American College of Emergency Physicians (ACEP) regarding the clinical use of resuscitative endovascular balloon occlusion of the aorta (REBOA). Trauma Surg. Acute Care Open.

[15] Lendrum R.A., Perkins Z., Marsden M., Cochran C., Davenport R., Chege F. (2024). Prehospital partial resuscitative endovascular balloon occlusion of the aorta for exsanguinating subdiaphragmatic hemorrhage. JAMA Surg..

[16] Madurska M.J., McLenithan A., Scalea T.M., Kundi R., White J.M., Morrison J.J. (2022). A feasibility study of partial REBOA data in a high-volume trauma center. Eur. J. Trauma Emerg. Surg..

[17] Gondek S., Hamblin S., Raley J., Nguyen J., Pandya U., Duchesne J. (2024). A PROMPT update on partial REBOA: initial clinical data and overview of the DoD-funded partial REBOA outcomes multicenter prospective (PROMPT) study. Mil. Med..

[18] Aaberg O.R., Johannesen D.T.S., Moi E.B., Frivold G., Jensen F., Haddeland K. (2025). Team leader communication in ad hoc teams and its impact on team outcomes: a systematic review. BMC Health Serv. Res..

[19] Theodrou C.M., Anderson J.E., Brenner M., Scalea T.M., Inaba K., Cannon J. (2020). Practice, practice, practice! Effect of resuscitative endovascular balloon occlusion of the aorta volume on outcomes: data from the AAST AORTA registry. J. Surg. Res..

